# Impact of *C57BL/6J* and *SV-129* Mouse Strain Differences on Ischemia-Induced Postnatal Angiogenesis and the Associated Leukocyte Infiltration in a Murine Hindlimb Model of Ischemia

**DOI:** 10.3390/ijms222111795

**Published:** 2021-10-30

**Authors:** Matthias Kübler, Philipp Götz, Anna Braumandl, Sebastian Beck, Hellen Ishikawa-Ankerhold, Elisabeth Deindl

**Affiliations:** 1 Walter-Brendel-Centre of Experimental Medicine, University Hospital, Ludwig-Maximilians-Universität München, 81377 Munich, Germany; Matthias.Kuebler@med.uni-muenchen.de (M.K.); P.Goetz@med.uni-muenchen.de (P.G.); Anna.Braumandl@med.uni-muenchen.de (A.B.); sebastian.beck@med.uni-muenchen.de (S.B.); Hellen.Ishikawa-Ankerhold@med.uni-muenchen.de (H.I.-A.); 2 Biomedical Center, Institute of Cardiovascular Physiology and Pathophysiology, Ludwig-Maximilians-Universität München, 82152 Planegg-Martinsried, Germany; 3 Department of Internal Medicine I, Faculty of Medicine, University Hospital, Ludwig-Maximilians-Universität München, 81377 Munich, Germany

**Keywords:** angiogenesis, *C57BL/6J* mice, *SV-129* mice, leukocytes, macrophages, neutrophils, NETs, neutrophil extracellular traps, *C57BL6*, 129S1/Sv

## Abstract

Strain-related differences in arteriogenesis in inbred mouse strains have already been studied excessively. However, these analyses missed evaluating the mouse strain-related differences in ischemia-induced angiogenic capacities. With the present study, we wanted to shed light on the different angiogenic potentials and the associated leukocyte infiltration of *C57BL/6J* and *SV-129* mice to facilitate the comparison of angiogenesis-related analyses between these strains. For the induction of angiogenesis, we ligated the femoral artery in 8–12-week-old male *C57BL/6J* and *SV-129* mice and performed (immuno-) histological analyses on the ischemic gastrocnemius muscles collected 24 h or 7 days after ligation. As evidenced by hematoxylin and eosin staining, *C57BL/6J* mice showed reduced tissue damage but displayed an increased capillary-to-muscle fiber ratio and an elevated number of proliferating capillaries (CD31^+^/BrdU^+^ cells) compared to *SV-129* mice, thus showing improved angiogenesis. Regarding the associated leukocyte infiltration, we found increased numbers of neutrophils (MPO^+^ cells), NETs (MPO^+^/CitH3^+^/DAPI^+^), and macrophages (CD68^+^ cells) in *SV-129* mice, whereas macrophage polarization (MRC1^-^ vs. MRC1^+^) and total leukocyte infiltration (CD45^+^ cells) did not differ between the mouse strains. In summary, we show increased ischemia-induced angiogenic capacities in *C57BL/6J* mice compared to *SV-129* mice, with the latter showing aggravated tissue damage, inflammation, and impaired angiogenesis.

## 1. Introduction

The terminal vessels of the vertebrate circulatory system are made up of arterioles, venules, and the capillary bed, with the latter being mandatory for maintaining the homeostasis of a living individual [1]. The anatomy and physiology of capillaries allow the direct exchange of gases, liquids, nutrients, signal molecules, and cells between blood and the adjacent tissue, thus ensuring proper tissue nourishment with oxygen and nutrients [1]. Furthermore, the regulation of capillary bed growth in the adult individual plays a vital role in physiological conditions as well as in many acute and chronic diseases, such as wound healing, cancer, and inflammation [2,3,4]. This process, denoted as angiogenesis, occurs either via capillary splitting or sprouting, leading to a more advanced capillary network structure accompanied by an amplification of the total capillary surface [5,6]. While, in cancer, uncontrolled capillary growth facilitates the nutrition and survival of tumor cells and their blood-dependent metastatic spread, in wound healing, impaired angiogenesis causes chronic stagnancy of the healing process. Thus, both promoting and inhibiting angiogenic processes are the object of different therapeutic approaches, dependent on the present pathology.

In vascular occlusive diseases, such as myocardial infarction and peripheral artery disease, the restoration of oxygen supply in the affected ischemic tissue downstream the occluded artery is only possible via the formation of natural bypasses, a process that is referred to as arteriogenesis [7,8]. Arteriogenesis describes the growth transformation of pre-existing collateral arteries, which is initially triggered by increasing fluid shear stress that leads to a local inflammatory process. This inflammation finally results in a caliber gain of the pre-existing interarterial anastomoses and therefore to a redirection of the blood flow, ensuring the appropriate re-supply of the ischemic tissue areas. Instead, angiogenesis does not occur to re-oxygenate the hypoxic tissue due to insufficient blood supply, but for cell debris removal and tissue reorganization in the ischemic muscle tissue areas [9,10,11].

The processes of angiogenesis are modified by a broad range of signaling molecules originating from a wide variety of cells. The most investigated pro-angiogenic factor is the vascular endothelial growth factor A (VEGFA) belonging to a family of strong pro-angiogenic regulators [1,12,13]. It is well described that VEGFA promotes endothelial cell differentiation, proliferation, and angiogenic remodeling via its binding to the receptor tyrosine kinase VEGF receptor 2 (VEGFR-2) [14,15]. VEGFA levels rise in an oxygen-dependent manner in hypoxic tissue [16]. However, VEGFA and other angiogenesis-modulating molecules, such as tissue modulating matrix metalloproteases (MMPs), are also distributed by leukocytes, such as neutrophils and macrophages [1,17,18]. Additionally, leukocytes directly affect angiogenesis by activating endothelial cells, remodeling the matrix, and stabilizing vessel anastomoses [17,18,19]. 

Thus, leukocytes play an essential role in controlling angiogenesis not only through the removal of cellular debris at the ischemic site but also through the remodeling of the surrounding matrix and tissue and the direct allocation of pro- and anti-angiogenic factors [1,19,20,21,22,23,24]. Consequently, the regulation of the inflammatory immune cell infiltration highlights a new therapeutic target to modulate the efficacy of the processes of angiogenesis.

In different experimental setups, different mouse strains are employed in murine hindlimb models of ischemia to evaluate the effects of different targets on arteriogenesis and angiogenesis. Following femoral artery ligation (FAL), collateral arteries in the adductor muscles in the upper leg grow to restore the hindlimb’s blood supply (arteriogenesis), while the provoked ischemia in the gastrocnemius muscle of the lower leg leads to hypoxia-dependent muscle tissue destruction and accompanied angiogenesis [25,26,27,28]. In the past, astonishing differences regarding the arteriogenic capacities between different inbred mouse strains have been observed, yet without analyzing the strain-related differences in angiogenesis and the associated leukocyte recruitment [29,30].

Two extremes of arteriogenic capacity analysis were marked by the two inbred mouse strains of the *C57BL/6J* line and the *SV-129* line, both widely used and well-established experimental inbred mouse strains. Comparing their arteriogenic capacities upon FAL, *C57BL/6J* mice showed the highest reperfusion rate, while *SV-129* mice showed attenuated reperfusion recovery [29].

It is important to notice that in the model of femoral artery ligation, the extent of angiogenesis highly depends on the efficacy of arteriogenesis; impaired collateral artery growth in the adductor muscle of the upper leg leads to increased ischemia in the distal gastrocnemius muscle of the lower leg and thus to a stronger ischemic trigger for the processes of angiogenesis [31]. So, for comparing ischemia-dependent angiogenesis upon FAL between different mouse strains, the associated arteriogenic capacities of these inbred lines have to be taken into account when interpreting the acquired data.

Inbred mouse strains are commonly accepted in research. Their genetic uniformity facilitates genetic research as the use of fewer individuals may lead to a statistical significance level. Having their origin in genetics and cancer research, the *C57BL6* line became the standard for most research applications, especially in cardiovascular research, since C. C. Little, the founder of Jackson Laboratory, established the line with mice from Abbie Lathrop about 100 years ago [32]. Due to the well-established embryonic stem cell line, *SV-129* mice are often used for targeted mutations, thus being the preferable mouse line concerning transgenic mouse strain design.

In the past, the yet unknown differences in ischemia-induced angiogenic efficacy between the two different inbred mouse strains of *C57BL/6J* and *SV-129* made it challenging to evaluate and compare findings in experiments conducted with these two strains. As *SV-129* mice show a decreased arteriogenic capacity, we hypothesized that a higher ischemic force might lead to a higher level of angiogenesis. The present study aimed to investigate whether this was the case and how the strains react pathophysiologically to femoral artery occlusion. Thus, with the present study, we shed light on the differences in ischemia-provoked angiogenic capacities and the accompanied leukocyte infiltration in a murine hindlimb model between the *C57BL/6J* and *SV-129* inbred mouse strains. 

## 2. Results

To compare the angiogenic potential of the two different mouse strains, we followed a well-established model of hindlimb ischemia: the right femoral artery of *C57BL/6J* and *SV-129* mice was occluded (FAL), leading to collateral growth (arteriogenesis) in the upper leg and angiogenesis in the lower leg on the occluded side, while the left leg underwent a sham operation [25]. At 24 h or 7 days after FAL, mice were sacrificed and the gastrocnemius muscles of the lower leg were collected for (immuno-) histological studies.

Monitoring the animal’s health in the experiment, we could not observe any strain-related differences concerning wound healing. No grave necrosis of the foot could be observed. As known, both strains regain their hindlimb function within 7 days after surgery [29]. A systemic assessment of the foot active use score was not performed in this study.

**Figure 1:**
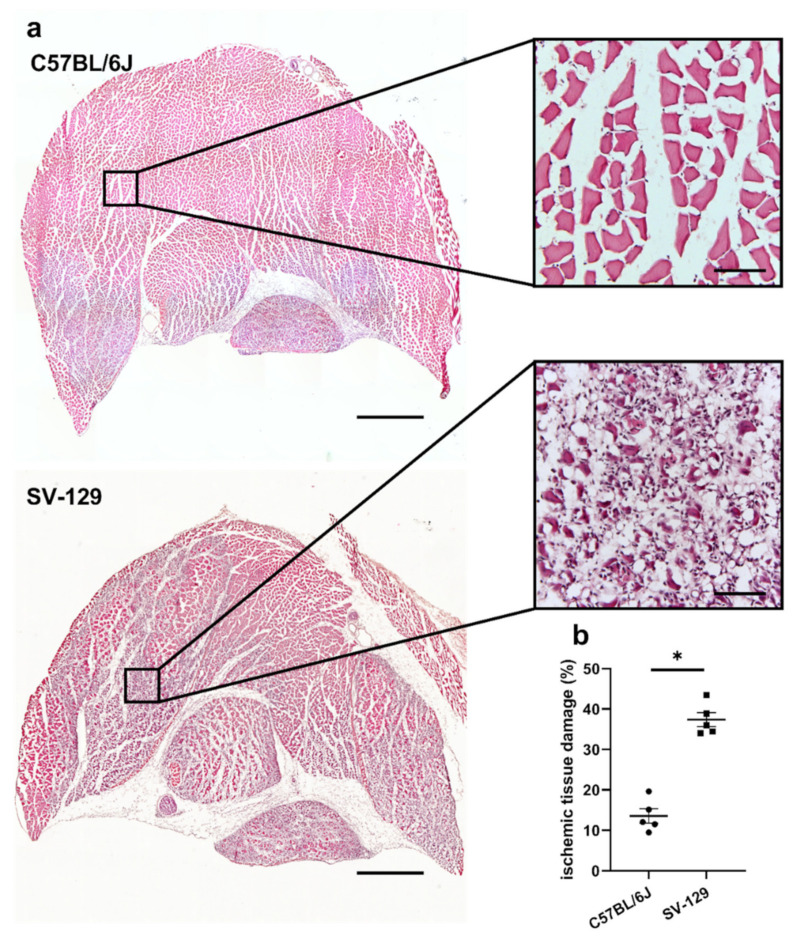
*C57BL/6J* mice show a smaller cross-sectional area of ischemic tissue damage in comparison to *SV-129* mice. (**a**) Representative pictures of hematoxylin and eosin (H&E)-stained gastrocnemius muscles of *C57BL/6J* (top) and *SV-129* mice (bottom) collected 7 days after femoral artery ligation (FAL). Skeletal muscle cells that show centralized nuclei are a sign of regenerating muscle cells and hence ischemic damage. Scale bars: 1000 µm (overview), 100 µm (detail). (**b**) The scatter plot displays the relative area of ischemic tissue damage (%) in gastrocnemius muscles of *C57BL/6J* and *SV-129* mice isolated 7 days after FAL. One complete sectional area was analyzed per mouse per group. Data are means ± S.E.M., *n* = 5 per group. * *p* < 0.05 (*C57BL/6J* vs. *SV-129*) by unpaired, two-sided Student’s *t*-test.

The area of ischemic damage in gastrocnemius muscles 7 days after FAL was analyzed using a hematoxylin and eosin (H&E) staining. *C57BL/6J* mice showed a decreased ischemic damage area compared to *SV-129* mice (Figure 1). Neither gastrocnemius muscles isolated from *C57BL/6J* nor *SV-129* mice showed any ischemic damage after sham operation (Figure S1).

**Figure 2:**
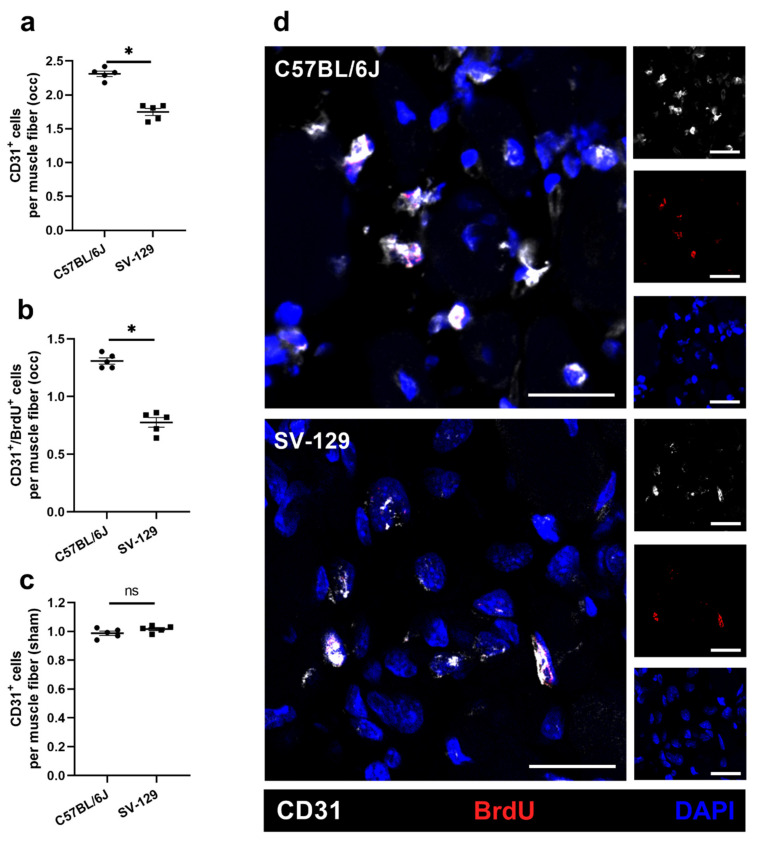
*C57BL/6J* mice show a higher capillarity under ischemic conditions than *SV-129* mice. The scatter plots display (**a**) endothelial cells (CD31^+^/DAPI^+^) per muscle fiber as well as (**b**) proliferating endothelial cells (CD31^+^/BrdU^+^ (bromodeoxyuridine)/DAPI^+^) per muscle fiber of *C57BL/6J* and *SV-129* mice in occluded (occ) ischemic gastrocnemius muscles isolated 7 days after femoral artery ligation (FAL). Scatter plot (**c**) displays the capillary-to-muscle fiber ratio of sham-operated (sham) non-ischemic gastrocnemius muscles isolated from *C57BL/6J* and *SV-129* mice 7 days after FAL. A defined ischemic area (1.5 mm^2^) of muscle tissue was analyzed per mouse. Data are means ± S.E.M., *n* = 5 per group. ^n.s.^ *p* ≥ 0.05, * *p* < 0.05 (*C57BL/6J* vs. *SV-129*) by unpaired, two-sided Student’s *t*-test. (**d**) Representative immunofluorescence staining of ischemic gastrocnemius muscles of *C57BL/6J* (top) and *SV-129* mice (bottom) collected 7 days after FAL. Cells were stained with an antibody labeling CD31 (white), BrdU (red), and DAPI (blue) to label nucleic DNA. Scale bars: 20 µm.

To measure the angiogenic capacity of the mouse strains under ischemic conditions, we stained gastrocnemius muscles for CD31/BrdU/DAPI and calculated the capillary-to-muscle fiber ratio from muscle tissue isolated 7 days after FAL. CD31 served as a capillary marker and bromodeoxyuridine (BrdU) served as a proliferation marker. As platelets also express CD31, only CD31^+^/DAPI^+^ signals were counted as capillary signals and quantified. In addition, CD31^+^/BrdU^+^/DAPI^+^ signals were quantified to investigate the number of proliferating capillaries. Compared to *SV-129* mice, *C57BL/6J* mice showed a higher number of capillaries and a higher number of proliferating capillaries per muscle fiber, indicating the increased angiogenic capacity of *C57BL/6J* mice (Figure 2a,b,d). To exclude any a priori differences in capillarity between the mouse strains, we analyzed non-ischemic gastrocnemius muscles, finding no significant differences in capillary-to-muscle fiber ratio (Figure 2c).

**Figure 3:**
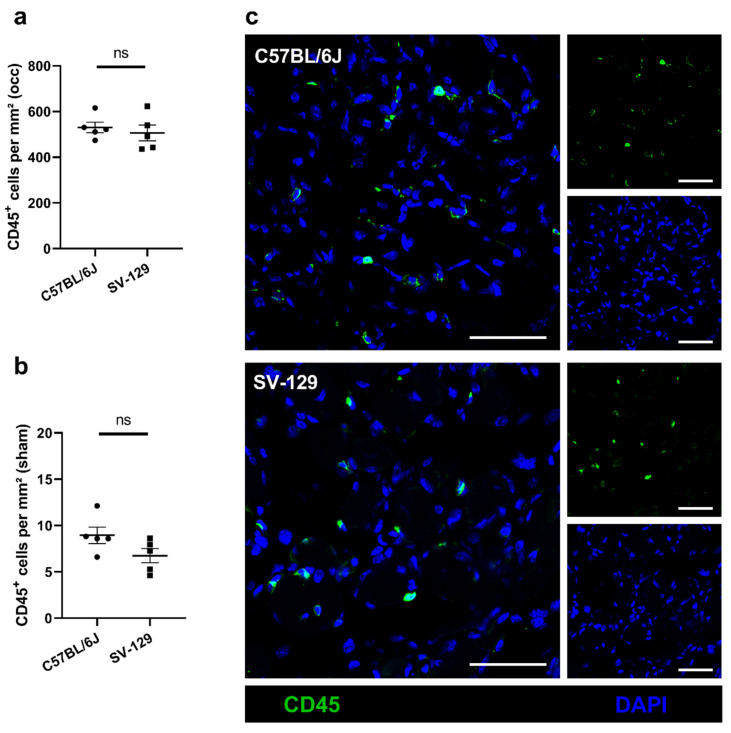
*C57BL/6J* and *SV-129* mouse strains show comparable leukocyte accumulation under ischemic conditions. The scatter plots display (**a**) the number of infiltrating leukocytes (CD45^+^/DAPI^+^) per mm^2^ in occluded (occ) ischemic gastrocnemius muscles of *C57BL/6J* and *SV-129* mice as well as (**b**) the number of leukocytes per mm^2^ in sham-operated (sham) non-ischemic tissue, all collected 7 days after femoral artery ligation (FAL). A defined ischemic area (1.5 mm^2^) of muscle tissue was analyzed per mouse. Data are means ± S.E.M., *n* = 5 per group. ^n.s.^ *p* ≥ 0.05, (*C57BL/6J* vs. *SV-129*) by unpaired, two-sided Student’s *t*-test. (**c**) Representative immunofluorescence staining of ischemic gastrocnemius muscles of *C57BL/6J* (top) and *SV-129* mice (bottom) isolated 7 days after FAL. Cells were stained with an antibody against CD45 (green) and DAPI (blue) to label nucleic DNA. Scale bars: 50 µm.

Leukocytes are known as regulators as well as enhancers of ischemia-induced inflammation, including cell debris removal and tissue regeneration, and directly influence vascular cell proliferation by their supply of growth factors. Consequently, we focused on changes in leukocyte accumulation related to the different mouse strains. Using CD45 as a pan-leukocyte marker, we quantified CD45^+^/DAPI^+^ signals in gastrocnemius muscles 7 days after surgery. Comparing both mouse strains, we did not find a significant difference between the *SV-129* and the *C57BL/6J* line (Figure 3a,c) in ischemic tissue samples. In addition, we observed no significant difference in the number of leukocytes in sham-operated muscles (Figure 3b).

**Figure 4:**
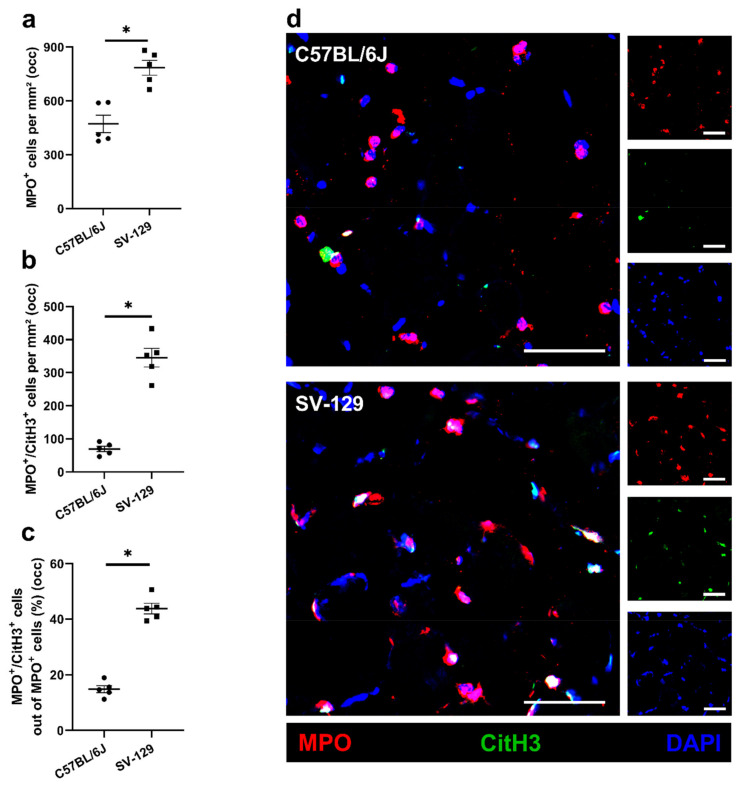
Compared to *SV-129* mice, accumulation of neutrophils and neutrophil extracellular trap (NET) formation is diminished in *C57BL/6J* mice under ischemic conditions. Scatter plots display the number of (**a**) neutrophils (MPO^+^ (myeloperoxidase)/DAPI^+^) per mm^2^, (**b**) neutrophil extracellular traps (MPO^+^/CitH3^+^ (citrullinated histone H3)/DAPI^+^) per mm^2^, and (**c**) the percentage of NETs/neutrophils in occluded (occ) ischemic gastrocnemius muscles isolated from *C57BL/6J* and *SV-129* mice 24 h after femoral artery ligation (FAL). A defined ischemic area (0.86 mm^2^) of muscle tissue was analyzed per mouse. Data are means ± S.E.M., *n* = 5 per group. * *p* < 0.05 (*C57BL/6J* vs. *SV-129*) by unpaired, two-sided Student’s *t*-test. (**d**) Representative immunofluorescence staining of ischemic gastrocnemius muscles of *C57BL/6J* (top) and *SV-129* mice (bottom) collected 24 h after FAL. Cells were stained with an antibody against MPO (red), CitH3 (green), and DAPI (blue) to label nucleic DNA. Scale bars: 50 µm.

To gain further information concerning leukocyte subpopulations with a focus on neutrophils and macrophages, we detected neutrophils and their formation of neutrophil extracellular traps (NETs) using a combined staining for myeloperoxidase (MPO) as a neutrophil marker and citrullinated histone H3 (CitH3) as a NET marker on tissue isolated 24 h after FAL. We found a significantly reduced number of neutrophils (MPO^+^/DAPI^+^), NETs (MPO^+^/CitH3^+^/DAPI^+^), and neutrophils, which are in the process of NET formation, in mice of the *C57BL/6J* strain in comparison to the *SV-129* line in ischemic tissue (Figure 4). Under non-ischemic conditions after sham operation, we could not observe differences in the number of neutrophils in tissue samples of both mouse strains (data not shown), while NETs were completely absent.

**Figure 5:**
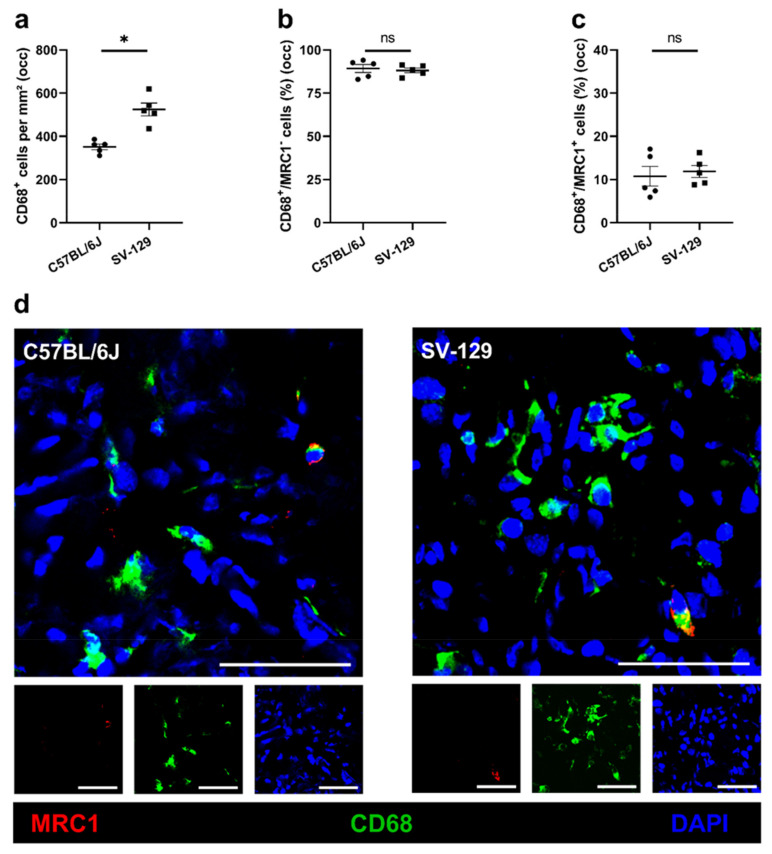
*C57BL/6J* mice show a lower number of macrophages than *SV-129* mice at the side of sterile, ischemic inflammation, while macrophage polarization is unaffected. Scatter plots display (**a**) the number of macrophages (CD68^+^/DAPI^+^) per mm^2^, (**b**) the percentage of M1-like polarized macrophages (CD68^+^/MRC1^−^ (mannose receptor C-type 1)/DAPI^+^), and (**c**) the percentage of M2-like polarized macrophages (CD68^+^/MRC1^+^/DAPI^+^) in occluded (occ) ischemic gastrocnemius muscles isolated from *C57BL/6J* and *SV-129* mice 7 days after femoral artery ligation (FAL). A defined ischemic area (1.5 mm^2^) of muscle tissue was analyzed per mouse. Data are means ± S.E.M., *nüber* = 5 per group. ^n.s.^ *p* ≥ 0.05, * *p* < 0.05 (*C57BL/6J* vs. *SV-129*) by unpaired, two-sided Student’s *t*-test. (**d**) Representative immunofluorescence staining of ischemic gastrocnemius muscles of *C57BL/6J* (left) and *SV-129* mice (right) collected 7 days after FAL. Cells were stained with an antibody against MRC1 (red), CD68 (green), and DAPI (blue) to label nucleic DNA. Scale bars: 50 µm.

Analyzing strain-dependent changes in macrophage accumulation and polarization in ischemic tissue 7 days after FAL, we used CD68 as a macrophage and mannose receptor C-type 1 (MRC1) as a macrophage polarization marker. CD68^+^/MRC1^-^ cells were counted as pro-inflammatory M1-like polarized macrophages and CD68^+^/MRC1^+^ cells as anti-inflammatory M2-like polarized macrophages accordingly. *C57BL/6J* mice displayed a lower infiltration of macrophages in ischemic tissue than *SV-129* mice, but no change in macrophage polarization was observed (Figure 5). In non-ischemic tissue, both mouse strains differed neither in macrophage accumulation nor macrophage polarization (data not shown).

## 3. Discussion

Researchers working with mouse models are often not aware of differences in the pathophysiological reactions of inbred mouse strains to experimental manipulations. In the case of experimental setups using FAL, the differences in the degree and the underlying molecular pathways of angiogenesis between inbred mouse strains have never been taken into account. Over the last few years, numerous studies on ischemia-induced angiogenesis using the well-established and important murine hindlimb model of FAL have been conducted using both the *C57BL/6J* and *SV-129* mouse strains. Yet, the angiogenic capacities of both inbred strains have never been compared, complicating the retrospective comparison and evaluation of already collected data [28,31,33,34]. With the present study, we performed a detailed investigation of *C57BL/6J* and *SV-129* mouse strains known for their different pathological reactions towards FAL, focusing on postnatal ischemia-induced angiogenesis and the associated leukocyte recruitment in the gastrocnemius muscle. 

Our results demonstrate that *C57BL/6J* mice show an increased capillary-to-muscle fiber ratio 7 days after FAL with an elevated number of proliferating capillaries and a reduced area of ischemia-induced tissue damage compared to *SV-129* mice. These findings were accompanied by a total reduction in infiltrating neutrophils (MPO^+^ cells), NETs (MPO^+^/CitH3^+^/DAPI^+^), and macrophages (CD68^+^ cells) in ischemic muscle tissue of *C57BL/6J* mice, while no significant differences in macrophage polarization and the total number of infiltrative immune cells (CD45^+^ cells) were observed. Altogether, our data suggest that *C57BL/6J* mice possessed better angiogenic capacities resulting in minor ischemic muscle tissue damage, probably caused by a milder, more pro-angiogenic inflammatory environment in *C57BL/6J* mice than in *SV-129* mice. 

Compared to *SV-129* mice, *C57BL/6J* mice showed a decreased area of ischemic tissue damage. In the mouse model of hindlimb ischemia, FAL results in the arteriogenic growth of collateral arteries in the upper leg and in ischemia-induced angiogenesis in the lower leg [25]. It was found that improved arteriogenesis in the upper leg results in a reduction in the ischemic tissue damage in the distal gastrocnemius muscle [31]. However, it is well described that the *C57BL/6J* strain has higher arteriogenic capacities than the *SV-129* strain, attributable to an improved increase in luminal diameter upon ligation [29]. Our results confirm these findings, as we found a decreased ischemic tissue damage area in *C57BL/6J* mice compared to *SV-129* mice, which is partly attributable to *C57BL/6J*’s improved arteriogenesis and the resulting higher perfusion of the lower limb.

Due to increased ischemia as the main angiogenic trigger, increased ischemic tissue damage is expected to be associated with enhanced angiogenesis. However, we found that despite the reduced tissue damage, *C57BL/6J* mice show a higher capillary-to-muscle fiber ratio and a higher number of proliferating capillaries per muscle fiber than *SV-129* mice. Accordingly, our results demonstrate that mice of the *C57BL/6J* strain show a more effective ischemia-induced angiogenesis than *SV-129* mice. Therefore, the observed decrease in ischemic tissue damage in *C57BL/6J* mice could also be a result of improved arteriogenesis, angiogenesis, and accompanied tissue reorganization.

In ischemic tissue damage, leukocytes migrate from the vasculature into the perivascular space to phagocyte damaged cells and initiate tissue reorganization comprising new capillary bed formation. Part of this tissue reorganization is creating local inflammation, leading to the release of other leukocyte-originating pro-inflammatory cytokines and chemokines. The allocation of these messenger substances results in further leukocyte recruitment at the damaged tissue area [17,18,35,36]. Additionally, this leukocyte migration is facilitated through a cytokine-dependent leakage of the endothelial cell barrier. After clearing the cellular debris and reorganizing the tissue, pro-inflammatory leukocytes leave the affected area, giving space for an anti-inflammatory and regenerating environment leading to tissue remodeling of the affected muscle tissue. While leukocytes, especially macrophages and neutrophils, are described to promote angiogenesis through the release of pro-angiogenic and tissue remodeling factors, excessive leukocyte accumulation is associated with the prolongation of infiltration and therefore decelerated healing processes [18,35,37]. However, we could not show a significant difference in the number of infiltrating leukocytes between both mouse strains.

In the earliest stages after the induction of FAL, neutrophils are recruited to the site of ischemic tissue damage, playing a pivotal role in initiating and modulating the local inflammatory and angiogenic processes [17]. By releasing potent forms of VEGFA, MMP-9, and other angiogenic factors, neutrophils are essential to promote the induction of angiogenesis as neutropenic mice failed to revascularize transplanted pancreatic island tissue [21,38,39,40]. Through their ability of phagocytosis, neutrophils participate in tissue repair and cell debris removal. Moreover, it was found that neutrophils form tunnels for vessel sprouts, supporting their growth and development in a model of thermal hepatic injury [37,41]. Nevertheless, neutrophils also release cytotoxic factors and excessive neutrophil accumulation is known to cause the aggravation of tissue damage [35,37,42]. 

Apart from that, neutrophil extracellular traps (NETs) are shown to promote angiogenesis in vivo and in vitro directly [43]. NETs are neutrophil-originated chromatin filaments with citrullinated histone H3 (CitH3) that are released into the extracellular space accompanied by various enzymes such as myeloperoxidase (MPO) as a reaction towards different inflammatory stimuli [44,45]. It was recently found that NETs promote angiogenesis through the modulation of intercellular adhesion molecule 1 (ICAM-1) expression on endothelial cells, thus affecting leukocyte migration out of the vasculature into the perivascular space and the modulation of VEGF signaling leading to changes in progenitor cell recruitment and endothelial cell migration [43]. Moreover, NETs are also described to participate in tissue remodeling by promoting apoptosis in senescent vasculature in retinopathy [46]. However, excessive NET accumulation at the inflammatory site is associated with the aggravation and general prolongation of inflammation [47,48]. 

We observed an increased number of neutrophils, NETs, and neutrophils forming NETs in the ischemic muscle tissue of mice belonging to the *SV-129* inbred strain. Thus, mice from the *C57BL/6J* strain show a milder, more pro-angiogenic inflammatory picture, whereas *SV-129* mice display aggravated inflammation in their ischemic muscle tissue one day after FAL.

In ischemic tissue, macrophages play a crucial role in supporting angiogenic tissue remodeling [18]. Hereby, their role depends on their plastic and changeable polarization state [49]. The shift in the macrophage polarization phenotype mirrors the shift from the pro- to the anti-inflammatory environment. The M1-like polarization (CD68^+^MRC1^-^) indicates a pro-inflammatory state of the macrophages, which shifts to an M2-like polarization (CD68^+^MRC1^+^), designating the change towards the subsequent regenerative anti-inflammatory phase of tissue restitution [18,50]. However, macrophages’ M1- and M2-like polarization states only reflect the extremes in a wide spectrum of macrophage polarization [49].

In the beginning, pro-inflammatory M1-like polarized macrophages are mainly responsible for clearing cellular debris through phagocytosis and promoting further local leukocyte recruitment via the allocation of pro-inflammatory chemoattractants [18]. Apart from that, M1-like polarized macrophages are described to support endothelial tip cell sprouting and to guide the growth of the newly formed vessels [51]. Furthermore, M1-like polarized macrophages are potent distributors of pro-angiogenic factors such as VEGFA and tumor necrosis factor-alpha (TNF-α) and are described to play an important role in angiogenesis as their depletion showed impaired angiogenic processes in zebrafish [51]. However, their impact on angiogenesis and tissue restitution is limited due to their restrained ability for matrix remodeling [18]. 

Afterward, the macrophages change their polarization towards an alternatively activated regenerative anti-inflammatory M2-like polarized phenotype [49]. The M2-like polarization state is mainly associated with tissue repair and the resolution of the previous inflammation [52,53,54,55]. In contrast to M1-like polarized macrophages, the M2-like polarized phenotype is essential for matrix remodeling. While in M1-like polarized macrophages, matrix remodeling proteases like matrix metalloproteinase 9 (MMP-9) are complexed and thus downregulated and inactive, in M2-like polarized macrophages, the complexation of these proteases is abolished and thus the enzymes can easily be released in their potent active form [56]. Thus, the M2-like polarized phenotype is denoted as the classic pro-angiogenic macrophage polarization state [18,57,58,59].

We found an increased number of infiltrating macrophages in the ischemic tissue of *SV-129* mice compared to *C57BL/6J* mice, while no differences in the ratio of M1- or M2-like proliferation state were detected. Nevertheless, the total number of M1- as well as M2-like polarized macrophages was higher in the ischemic muscle tissue of *SV-129* mice. Thus, the difference between the inbred mouse strains had no impact on macrophage polarization but on the number of infiltrating macrophages. Even though we found more pro-angiogenic M2-like polarized macrophages in the ischemic muscle tissue of *SV-129* mice, they did not show ameliorated angiogenesis compared to *C57BL/6J* mice. Accordingly, the increased number of infiltrative macrophages, especially the M1-like polarized phenotype, in *SV-129* mice could mirror an aggravated and prolonged inflammatory state associated with the increased ischemic tissue damage found in this mouse strain.

Moreover, we want to mention that the spatial distribution of macrophage subpopulation is well investigated in the process of arteriogenesis. C. Troidl et al. have observed a rising number of both M1- and M2-like polarized macrophages in the perivascular space until 28 days after ligation [60]. Interestingly, M1-like polarized macrophages were most likely found in the media, while M2-like polarized macrophages were found in the adventitia of the vessels, leading to the conclusion that they might fulfill different distinct roles in arteriogenesis. In contrast to the findings in collateral growth, in angiogenesis we could observe a predominantly pro-inflammatory M1-like polarized phenotype at 7 days after surgery with a spatial distribution throughout all the affected ischemic muscle tissue.

Altogether, we could show that *C57BL/6J* mice show improved angiogenesis after the onset of ischemia compared to mice from the *SV-129* strain. This difference in increased angiogenic growth capacity in *C57BL/6J* mice might be partly attributable to a milder, more pro-angiogenic inflammation based on reduced numbers of infiltrative macrophages, neutrophils, and NETs as compared to *SV-129* mice.

Concerning their specific impact, angiogenic factors and receptors have been extensively studied in various in vitro and in vivo experimental settings and reviewed [1]. The quantitative amounts of individual angiogenic factors alone are not exclusively responsible for modulating angiogenesis. Instead, the activation state of receptors, the number of co-receptors, and the activation status of co-receptors are as important as the number of angiogenic factors alone [61]. To gain a better overall understanding of the angiogenic capacities and their mediation in *C57BL/6J* and *SV-129* mice, we representatively analyzed the present immune microenvironmental conditions. However, further in-depth studies are necessary to evaluate the possible differences of other angiogenesis-modulating factors in *C57BL/6J* and *SV-129* mice.

With the present study, we aimed to advance the field of angiogenesis research by allowing a retrospective comparison and evaluation of already conducted studies on angiogenesis that used either *C57BL/6J* or *SV-129* mice. Furthermore, as researchers are often unaware of the importance of the appropriate mouse strain choice for their experimental setups, disregarding the differences in the pathophysiological reactions of inbred mouse strains to experimental manipulations, we also tried to point out the importance of mouse strain choice for experimental setup design. In synopsis with the presented immunological analysis concerning leukocyte infiltration and inflammation, we could show the detrimental differences between *C57BL/6J* and *SV-129* mouse strains for FAL-induced angiogenesis. Thus, evaluating the applicability of mouse strain choice regarding the research’s question for the planned experimental setup before conducting the experiments is still a very important and crucial part in experimental design.

## 4. Materials and Methods

### 4.1. Animals and Treatments

Experimental setups and animal care were permitted by the Bavarian Animal Care and Use Committee (ethical approval code: ROB-55.2Vet-2532.Vet_02-17-99, approved on the 8 December 2017) and were performed in strict accordance with the German animal legislation guidelines. Mice were housed in a temperature-controlled room on a 12-h light-dark cycle and received a standard laboratory diet. For all investigations, adult male *C57BL/6J* and 129S1/Sv (*SV-129*) (both Charles River Laboratories, Sulzfeld, Germany) mice, aged 8–12 weeks, were sacrificed at 24 h or 7 days (per timepoint *n* = 5 per group) after FAL. For determining the proliferation rate of endothelial cells in the gastrocnemius muscle 7 days after surgery, mice received a daily injection of 100 µL BrdU (Sigma-Aldrich, St. Louis, MO, USA) (12.5 mg/mL BrdU in PBS (PAN Biotech)) i.p., starting directly after the surgical intervention.

### 4.2. Femoral Artery Ligation and Tissue Processing

To initiate the processes of angiogenesis in the gastrocnemius muscle of the lower hindlimb, the right femoral artery was unilaterally ligated while the left hindlimb was sham-operated and served as an internal control, as previously described [25]. Before the ligation, mice were anesthetized with a s.c. injection of fentanyl (0.05 mg/kg, CuraMED Pharma, Karlsruhe, Germany), midazolam (5.0 mg/kg, Ratiopharm GmbH, Ulm, Germany), and medetomidine (0.5 mg/kg, Pfister Pharma, Berlin, Germany). Before tissue collection 24 h or 7 days after FAL, mice were again anesthetized as described above. After sacrificing, the hindlimbs were perfused with a combination of adenosine buffer (1% adenosine (Sigma-Aldrich, St. Louis, MO, USA), 5% bovine serum albumin (BSA, Sigma-Aldrich, St. Louis, MO, USA, dissolved in PBS) and 3% (for cryopreservation) paraformaldehyde (PFA, Merck, Darmstadt, Germany, dissolved in PBS). For immunohistology, the ligated and the sham-operated hindlimbs of each mouse were collected after the perfusion, then embedded in Tissue-Tek compound (Sakura Finetek Germany GmbH, Staufen, Germany), and finally cryopreserved at −80 °C for further analysis.

### 4.3. Histology and Immunohistology

For (immuno-) histological staining, the cryopreserved gastrocnemius muscles were cut in 10-µm-thick slices. Tissue collected 7 days after FAL was used for immunohistological staining of endothelial cells, leukocytes, and macrophages as well as H&E staining, whereas neutrophils and NETs were analyzed on gastrocnemius muscles collected 1 day after ligation.

For the labeling of proliferating cells, the BrdU-treated tissue was incubated with 1 N HCl in a humidified chamber at 37 °C for 30 min, then permeabilized with a 0.2% Triton X-100 solution (AppliChem GmbH, Darmstadt, Germany) in 1 × PBS/0.1% Tween-20 (AppliChem GmbH, Darmstadt, Germany)/0.5% BSA for 2 min, followed by blocking with 10% goat serum (Abcam, ab7481, Cambridge, UK) in 1 × PBS/0.1% Tween-20/0.5% BSA for 1 h at room temperature (RT). Subsequently, the BrdU-treated muscle tissues were incubated with the primary anti-BrdU-antibody (Abcam, ab6326, dilution 1:50 in 10% goat serum, Cambridge, UK) at 4 °C overnight. Following the next day, the cryosections were labeled with a secondary goat anti-rat Alexa Fluor^®^-546 antibody (Invitrogen, Thermo Fischer Scientific, A-11081, Carlsbad, CA, USA, dilution 1:100) for 1 h at RT. After secondary blocking with 1 × PBS/0.1% Tween-20/4% BSA for 30 min at RT, we applied an anti-CD31-Alexa Fluor^®^ 647 antibody (Biolegend, 102516, San Diego, CA, USA, dilution 1:50 in 1 × PBS/0.1% Tween-20) for labeling endothelial cells for 2 h at RT. 

For macrophage labeling, we used an anti-CD68-Alexa Fluor^®^ 488 antibody (Abcam, ab201844, dilution 1:200 in PBS, Cambridge, UK) together with a primary anti-MRC1 antibody (Abcam, ab64693, dilution 1:200 in PBS, Cambridge, UK) to ascertain macrophage polarization and incubated both at 4 °C overnight. Secondary antibody staining was conducted with a donkey-anti-rabbit Alexa Fluor^®^ 546 antibody (Invitrogen, A-10040) for 1 h at RT.

For NETs staining, cryosections collected 24 h after FAL were initially permeabilized with 0.2% Triton X-100 solution in 1 × PBS/0.1% Tween-20/0.5% BSA for 2 min, then blocked with 10% donkey serum (Abcam, ab7475, Cambridge, UK) in 1 × PBS/0.1% Tween-20/0.5% BSA for 1 h at RT followed by incubation with the primary antibodies anti-myeloperoxidase (MPO; R&D Systems, AF3667, Minneapolis, MN, USA, dilution 1:20 in 10% donkey serum in 1 × PBS/0.1% Tween-20/0.5% BSA) and anti-CitH3 antibody (polyclonal rabbit anti-Histone H3 (citrulline R2+R8+R17), Abcam (Cambridge, UK), ab5103, dilution 1:100 in 10% donkey serum in 1 × PBS/0.1% Tween-20/0.5% BSA) at 4 °C overnight. A donkey anti-goat Alexa Fluor^®^ 594 (Invitrogen, A-11058, dilution 1:100 in 1 × PBS/0.1% Tween-20) and a donkey anti-rabbit Alexa Fluor^®^ 488 antibody (Invitrogen, A-21206, dilution 1:200 in 1 × PBS/0.1% Tween-20) were used for secondary antibody staining for 1 h at RT. 

Additionally, DAPI (Thermo Fisher Scientific, Waltham, MA, 62248, dilution 1:1000 in PBS) was co-incubated on all cryosections for nucleic DNA labeling for 10 min at RT. The stained tissue sections were finally mounted with an antifade mounting medium (Dako, Agilent, Santa Clara, CA, USA). H&E staining was conducted according to the manufacturer’s instruction (Carl Roth GmbH, Karlsruhe, Germany).

For microscopic imaging, we employed a confocal laser scanning microscope LSM 880 (Carl-Zeiss Jena GmbH, Jena, Germany) with a 20× objective (415 µm × 415 µm) as well as an epifluorescence microscope (Leica DM6 B, Leica microsystems, Wetzlar, Germany) with a 20× objective (630 µm × 475µm). For each muscle section, we imaged 5 defined fields to quantify cells, muscle fibers, and NETs. To ascertain the areas of damaged tissue (%), the total gastrocnemius muscle area was analyzed. Gastrocnemius sections stained with H&E, CD45/DAPI, and CD31/BrdU/DAPI were analyzed with the epifluorescence microscope. CD68/MRC1/DAPI and MPO/CitH3/DAPI labeled muscle sections were investigated with the confocal laser scanning microscope. Cell quantification and analysis of the damaged muscle area were conducted using ImageJ software. We calculated the capillary-to-muscle fiber ratio (CD31^+^/DAPI^+^ cells were counted as endothelial cells) as described before to assess the processes of angiogenesis [62]. 

### 4.4. Statistical Analysis

Statistical analyses were performed and graphically outlined with GraphPad Prism 8 (GraphPad Software, La Jolla, CA, USA). Data are means ± standard error of the mean (S.E.M.). Statistical analyses were calculated as described in the figure legends. Results were considered as statistically significant at *p* < 0.05. 

## Data Availability

The data presented in this study are available on request from the first authors.

## Supplementary Materials

The following are available online at https://www.mdpi.com/article/10.3390/ijms222111795/s1.
