# Supplementary material for: Impact of C57BL/6J and SV-129 Mouse Strain Differences on Ischemia-Induced Postnatal Angiogenesis and the Associated Leukocyte Infiltration in a Murine Hindlimb Model of Ischemia
Source: Int J Mol Sci. 2021 Oct 30;22(21):11795. doi: 10.3390/ijms222111795 (PMC8584150; doi:10.3390/ijms222111795)
Supplement: Supplementary file 1 [file ijms-22-11795-s001.zip › ijms-1410173-supplementary.pdf]

International Journal of *Molecular Sciences*

Article

Impact of C57BL/6J and SV-129 mouse strain differences on ischemia-induced postnatal angiogenesis and the associated leukocyte infiltration in a murine hindlimb model of ischemia

Matthias Kübler [1,2,†], Philipp Götz [1,2,†], Anna Braumandl [1,2], Sebastian Beck [1,2], Hellen Ishikawa-Ankerhold [1,3] and Elisabeth Deindl [1,2,*]

1 Walter-Brendel-Centre of Experimental Medicine, University Hospital, Ludwig-Maximilians-Universität München, 81377, Munich, Germany; Matthias.Kuebler@med.uni-muenchen.de (M.K.); P.Goetz@med.uni-muenchen.de (P.G.); Anna.Braumandl@med.uni-muenchen.de (A.B); sebastian.beck@med.uni-muenchen.de (S.B.); Hellen.Ishikawa-Ankerhold@med.uni-muenchen.de (H.I.-A.); Elisabeth.Deindl@med.uni-muenchen.de (E.D.)
2 Biomedical Center, Institute of Cardiovascular Physiology and Pathophysiology, Ludwig-Maximilians-Universität München, 82152, Planegg-Martinsried, Germany
3 Department of Internal Medicine I, Faculty of Medicine, University Hospital, Ludwig-Maximilians-Universität München, 81377, Munich, Germany
* Correspondence: Elisabeth.Deindl@med.uni-muenchen.de; Tel.: +49 (0) 89 2180 76504
† These authors contributed equally to this work.

Citation: Kübler, M.; Götz, P.; Braumandl, A.; Beck, S.; Ishikawa-Ankerhold, H.; Deindl, E. Impact of C57BL6/Jand SV-129 mouse strain differences on ischemia-induced postnatal angiogenesis and the associated leukocyte infiltration in a murine hindlimb model of ischemia. *Int. J. Mol. Sci.* **2021**, *22*, 1795. https://doi.org/10.3390/ijms222111795

Academic Editor: Maria Luisa Balestrieri

Received: 21 September 2021
Accepted: 27 October 2021
Published: 30 October 2021

Publisher's Note: MDPI stays neutral with regard to jurisdictional claims in published maps and institu-tional affiliations.

Copyright: © 2021 by the authors. Submitted for possible open access publication under the terms and conditions of the Creative Commons Attribution (CC BY) license (http://creativecommons.org/licenses/by/4.0/).

Supplement

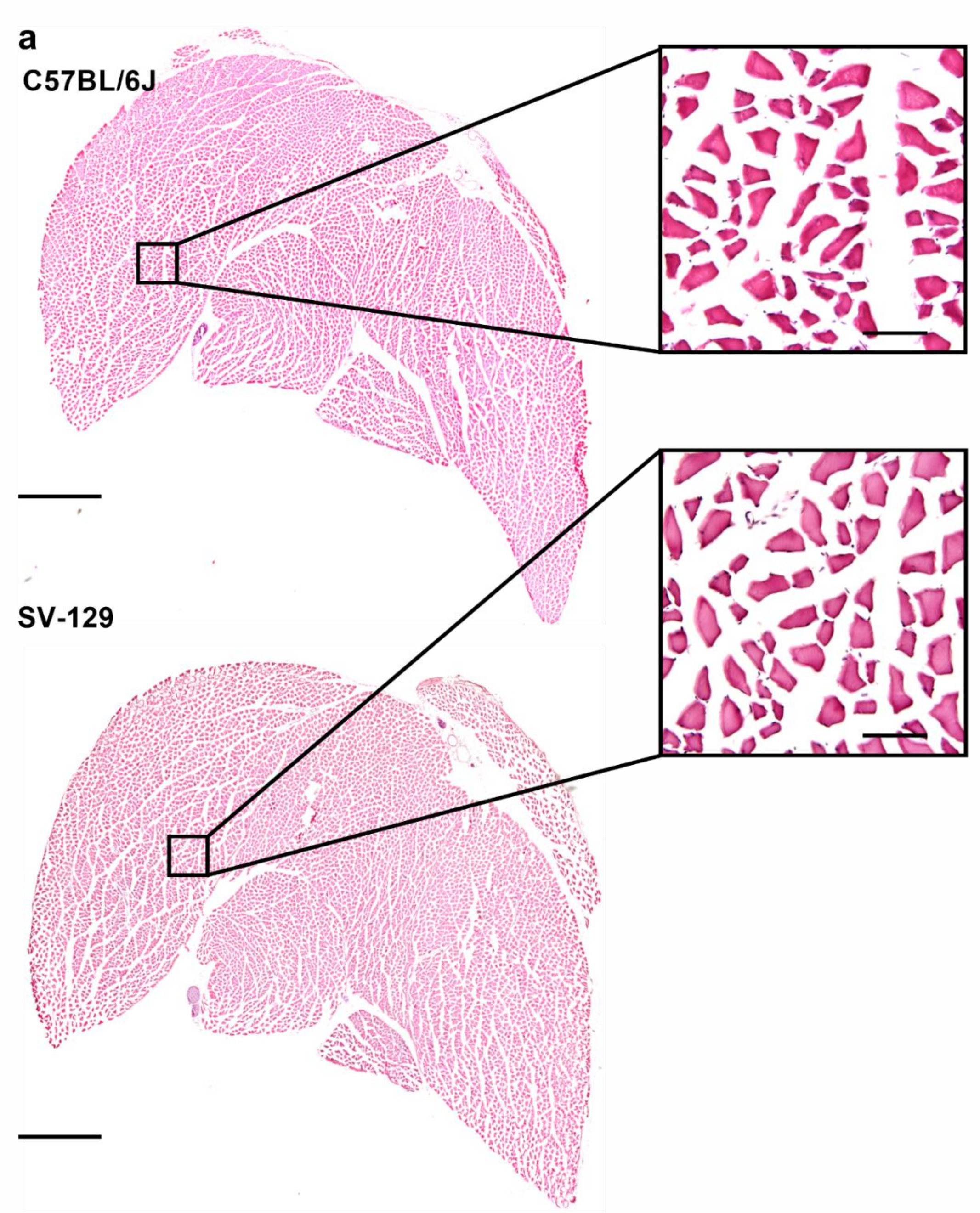

Supplement Figure S1. Gastrocnemius muscle tissue from sham-operated *C57BL/6J* and *SV-129* mice show no ischemic tissue damage. Representative pictures of hematoxylin and eosin (H&E)-stained sham-operated gastrocnemius muscles of *C57BL/6J* (top) and *SV-129* mice (bottom) collected 7 days after femoral artery ligation (FAL). Scale bars: 1000 μm (overview), 100 μm (detail).
